# Development and Evaluation of a Human Skin Equivalent in a Semiautomatic Microfluidic Diffusion Chamber

**DOI:** 10.3390/pharmaceutics13060910

**Published:** 2021-06-20

**Authors:** Júlia Tárnoki-Zách, Elod Mehes, Zsófia Varga-Medveczky, Dona Greta Isai, Nandor Barany, Edina Bugyik, Zsolt Revesz, Sándor Paku, Franciska Erdo, Andras Czirok

**Affiliations:** 1 Department of Biological Physics, Eotvos University, 1117 Budapest, Hungary; zachjuli@yahoo.fr (J.T.-Z.); emehes@caesar.elte.hu (E.M.); 2 Faculty of Information Technology and Bionics, Pázmány Péter Catholic University, 1083 Budapest, Hungary; varga-medveczky.zsofia@itk.ppke.hu; 3 Department of Anatomy and Cell Biology, University of Kansas Medical Center, Kansas City, KS 66160, USA; donnagreta@gmail.com; 4 First Department of Pathology and Experimental Cancer Research, Semmelweis University, 1085 Budapest, Hungary; nandorbarany@gmail.com (N.B.); bedush@gmail.com (E.B.); paku.sandor@med.semmelweis-univ.hu (S.P.); 5 Revesz Plasztika, 1125 Budapest, Hungary; revesz.zsolt@dpckorhaz.hu

**Keywords:** skin equivalent, electrospun mesh, microfluidic diffusion chamber, transepithelial transport kinetic, 3D printed device

## Abstract

There is an increasing demand for transdermal transport measurements to optimize topical drug formulations and to achieve proper penetration profile of cosmetic ingredients. Reflecting ethical concerns the use of both human and animal tissues is becoming more restricted. Therefore, the focus of dermal research is shifting towards in vitro assays. In the current proof-of-concept study a three-layer skin equivalent using human HaCaT keratinocytes, an electrospun polycaprolactone mesh and a collagen-I gel was compared to human excised skin samples. We measured the permeability of the samples for 2% caffeine cream using a miniaturized dynamic diffusion cell (“skin-on-a-chip” microfluidic device). Caffeine delivery exhibits similar transport kinetics through the artificial skin and the human tissue: after a rapid rise, a long-lasting high concentration steady state develops. This is markedly distinct from the kinetics measured when using cell-free constructs, where a shorter release was observable. These results imply that both the established skin equivalent and the microfluidic diffusion chamber can serve as a suitable base for further development of more complex tissue substitutes.

## 1. Introduction

Topical drug administration has many advantages over per os or systemic treatments. Locally applied active compounds can be used in lower doses, often can act locally or systemically without significant adverse effects, and bypass the first pass metabolism in the liver. Several drugs are formulated as a cream, ointment, solution, suspension or even transdermal patches and are applied to the skin surface. The target effect can be dermatological (anti-inflammatory, antibiotic, anti-allergic) but topical medication may also target the central nervous system as in the case of morphine [1] or nicotine patches [2]. Several humoral therapies are also applied on the skin [3] to reduce the peripheral side effects.

In vivo animal models were widely used to test the toxicity or local irritancy of drugs, chemicals and drug formulations. In pharmaceutical testing of dermal drugs the most widely used equipment is a diffusion cell—a device which is used in various configurations: horizontal [4], vertical [5], static [6] or flow-through [7]. In the last decade diffusion cells were miniaturized and skin-on-a-chip devices designed, developed and fabricated (for review see [8]). These microfluidic platforms still frequently utilize excised skin samples [9,10], but organotypic cultures could be also used [11]. Some companies already started to develop ex vivo and in vitro systems to mimic skin physiology using cell cultures of keratinocytes, fibroblasts and melanocytes. The preparation of such skin substituents may involve new technologies such as electrospinning where various scaffolds are prepared to ensure the optimal mechanical properties of the artificial tissues [12] or 3D bioprinting [13]. These novel technologies could revolutionize not only toxicological testing but the regenerative therapy of skin diseases, burn injury and wound healing.

Ethical considerations also favor the use of skin-on-a-chip models and skin equivalents. Several countries prohibit testing of cosmetics in animals or in tissues with animal origin. In particular, the EU Directive 76/768/EEC (2004) states that Scientists in both universities and industry must be educated, trained and supported, to develop the skills to make the change to alternative, human-based testing and research systems where they are available. The acceptance and validation of non-animal methods must evolve to ensure new techniques are approved and implemented quickly [14]. These regulations as well as compliance to the 3R rules (reduction, replacement and refinement of animal experiments) in cosmetic and drug testing makes it increasingly necessary to use artificial skin substituents to determine the efficacy and safety of formulations [15,16,17,18,19]. In this manuscript we present a simple measurement system consisting of a skin equivalent and a microfluidic diffusion chamber device. As a proof-of-concept study, we compare caffeine transport between the skin equivalent and human skin samples. We show that the presence of living cells is required to provide a transepithelial transport kinetics similar to that observed in human skin samples.

## 2. Materials and Methods

### 2.1. Electrospun Membranes

Polycaprolactone (PCL, Mw 70,000–90,000, 2-Oxepanone homopolymer, 6-Caprolactone polymer, Cat No.: 440744-500G) was purchased from Sigma-Aldrich. Formic Acid (88%, Cat No.: A118P-500) and Acetic Acid, Glacial (Cat No.: BP2401-500) were purchased from Fisher Chemical. PCL pellets were dissolved in a 1:1 (*v*/*v*) mixture of acetic acid and formic acid to yield 10 mL 15 *w*/*v*% solution based on a systematic published study [20]. The solution was magnetically stirred for at least 3 h to completely dissolve the pellets and to get a homogeneous solution. The solution was used within 3 h after the solution was ready. The prepared PCL solution was placed to in a 6-mL polypropylene syringe with a 18 G needle gauge of 1.20 mm. The feed rate of the polymer solution was 1 mL/h, which was provided by a syringe pump. The electrospinning voltage (26 kV) was applied to the needle at room temperature using a DC power supply. Electrospun meshes were collected on a 178 × 100 mm aluminum foil (Reynolds Wrap, heavy duty) pieces, at a distance of 23 cm from the needle. The thickness of the membrane can be controlled by the collection time: a 3 h long collection yields a 100 µm thick mesh weighing 150 mg.

### 2.2. 3D Printed Sample Holders

Both the base and ring element of the sample holder device were fused filament deposition- (3D-) printed using polylactic acid filament (PLA, Ultimaker B.V., Utrecht, The Netherlands) and an Ultimaker Original+ printer. The base part contains a 6 mm wide and 2.5 mm tall cylinder open at both ends. The cylinder is joined to a concentric disk (0.5 mm tall, 12 mm wide) with a 5.5 mm diameter inner hole. An 8 mm wide electrospun membrane patch is stretched and held on the top of the sample holder by a 3 mm tall cone shaped ring, with 6.2 mm and 6 mm bottom and top diameters, respectively. The assembled sample holder device is disinfected in 70% ethanol for 20 min, then dried under a sterile hood. The core of the sample holder device was filled with 75 µL collagen-I gel (1.7 mg/mL, Corning, New York, NY, USA, Cat No: 354236), prepared according to the manufacturer’s protocol. Briefly, collagen-I dissolved in 20 mM acetic acid (Corning) was neutralized with 1M NaOH and 10× PBS (Lonza, Basel, Switzerland) and transferred to the device at 37 °C where gelation was completed in 30 min. Finally, the electrospun mesh was coated by incubation with 5 µg/mL fibronectin (Sigma, St. Louis, MO, USA, Cat No: F1141) in PBS for 5 h at room temperature. The sample holder device was incubated in cell culture medium in the CO${}_{2}$ incubator at 37 °C for 2 h before use.

### 2.3. Cell Culture

HaCaT immortalized human keratinocytes were obtained from Cell Lines Service and cultured in DMEM medium (Lonza, Cat No.: 12-604F) supplemented with 10% FBS (Gibco Thermo Fisher, Waltham, MA, USA). Cultures were maintained in 6-well culture plates (Greiner, Frickenhausen, Germany) at 37 °C in a humidified incubator with 5% CO${}_{2}$ atmosphere. Once confluent, cell monolayers were washed in phosphate-buffered saline (PBS) twice, briefly incubated in trypsin-EDTA (Lonza), then resuspended in culture medium and transferred to the sample holder device at 1200 cells/mm${}^{2}$ density. Cells attached to the fibronectin-coated electrospun membrane and formed a confluent monolayer. Cultures were maintained for various durations up to 3 weeks while the sample holder devices were kept in standard 24-well tissue culture plates (Greiner) and medium was refreshed every 3 days.

### 2.4. Cell Labeling and Viability

To monitor the health of the cultures and to live-stain the cells, the live/dead viability kit (Thermo Fisher, Waltham, MA, USA, Cat No.: L3224) was used according to manufacturer’s protocol. Briefly, green-fluorescent calcein-AM was applied in 2 μM concentration for 30 min to indicate intracellular esterase activity of live cells by conversion to a fluorescent compound in their cytoplasm. After removal of the calcein, the red-fluorescent ethidium homodimer-1 was used at a concentration of 8 μM for 20 min to indicate loss of plasma membrane integrity in dead cells where nuclei were labeled. Cell cultures double-labeled this way were imaged with epifluorescence microscopy, or fixed with 4% paraformaldehyde in PBS and transferred to cryo-sectioning.

### 2.5. Histology

For histology, cell cultures maintained on electrospun membrane were fixed with 4% paraformaldehyde in PBS. Samples were embedded in Cryomatrix resin (Thermo Fisher) and cryo-sectioned using a Cryostar NX50 cryostat (Thermo Scientific) to obtain 14 μm thick sections. The samples were mounted on microscopic slides (Thermo Scientific) in Prolong mounting medium with NucBlue counterstain (Thermo Fisher) and imaged with phase contrast and epifluorescence microscopy. For semi-thin sections cell cultures were fixed in 4% paraformaldehyde/1% glutaraldehyde. Samples were dehydrated in a graded series of acetone, and embedded in Spurr’s resin (Sigma). Semi-thin sections were cut with a Reichert Ultracut microtome, mounted on microscopic slides (Thermo Scientific), stained with toluidine blue and imaged with brightfield microscopy.

### 2.6. Microscopy

Epifluorescence, phase-contrast or brightfield imaging was performed on a Zeiss Axio Observer Z1 inverted microscope equipped with 40× EC Plan-Neofluar objective, Colibri illumination system and Zeiss AxioCam MRm CCD camera. Images were processed using NIH ImageJ software.

### 2.7. Human Skin Samples

Human abdominal skin samples were provided by Révész Plasztika (plastic surgery clinics) and were used based on the permission for experimental application of human tissue No. 6501-6/2019/EKU. The excised skins were cleaned from subcutaneous adipose tissue and then stored frozen wrapped in an aluminium foil at −80 °C. On the day of the transdermal transport experiments the human tissue was thawed, mechanically sensitized with 30 fold tape strippings by an adhesive tape and cut into appropriate size to fit to the donor chamber of the microfluidic diffusion chamber device.

### 2.8. Microfluidic Diffusion Cell Device

Similarly to the traditional Franz-diffusion cell system, the polydimethylsiloxane-based microfluidic chip is also composed of three functional elements: a donor compartment where the experimental formulation is placed, the receptor compartment beneath the sample. The two compartments are separated by a skin sample or skin equivalent [9]. The diffusion surface of the sample was 1.766 cm${}^{2}$ and it was treated with 1000 μL cream formulation by a Microman E piston gel pipette (Gilson). Contrary to generally used Franz-diffusion cells, in the microfluidic diffusion chamber provides a continuous flow of the perfusion fluid below the treated skin surface at the subcutaneous area. For studies executed on human skins peripheral perfusion fluid (PPF, 147 mM NaCl, 4 mM KCl, and 2.3 mM CaCl${}_{2}$·2H${}_{2}$O) was used as an artificial extracellular fluid acceptor solution. For studies performed with skin substituents (HaCaT cell culture on mesh) cell culture medium was used as a perfusion and acceptor solution. The perfusion medium was loaded into a 5 mL syringe which was connected to a programmable syringe pump. Air bubbles were carefully removed from the syringe and tubes, and also from the microchannel of the chip. Flow rate was kept at 4 μL/min during the experiments. Collected samples were stored at −80 °C until spectrophotometric analysis.

### 2.9. Spectrophotometry

Caffeine content of the perfusion medium samples was determined using a NanoDrop 2000 system (Thermo Scientific) at 273 nm wavelength using 2 μL volumes. We used a gradually diluted sequence of caffeine solutions as a calibration curve to determine sample concentrations from the absorbance values.

### 2.10. Topical Formulation

A cream containing 2% suspension of caffeine (Sigma) was used in all studies. The cream consists of paraffin oil (Semmelweis University, University Pharmacy, Budapest, Hungary) 4.1%, vaseline ointment 47%, propylenglycol (Hungaropharma, Budapest, Hungary) 10%, 0.547%-citric acid solution 14% and purified water 22.9%. The composition of the vaseline ointment includes polysorbate (Hungaropharma, Budapest, Hungary) 4%, paraffin oil 8%, vaselinum album (Hungaropharma, Budapest, Hungary) 26%, alcohol cetylstearylicum (Molarchemicals, Halásztelek, Hungary) 12%, propylenglycol 10% and purified water 40%.

## 3. Results

### 3.1. Skin Equivalent in a Sample Holder Device

**Figure 1:**
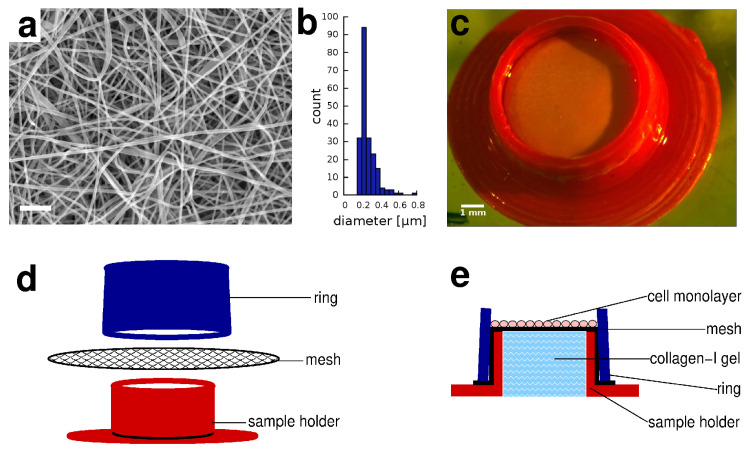
Skin equivalent sample holder device. (**a**) Scanning electron microscopic image of the electrospun PCL nanomesh consisting of filaments with sub-micron diameter. Scale bar: 3 μm. (**b**) Filament diameter distribution of the electrospun nanomesh, obtained from n=4 independent samples. (**c**) The assembled sample holder device, scale bar represents 1 mm. (**d**) Schematic drawing depicting the parts of the device. (**e**) Schematic cross section of the assembled sample holder device. The electrospun nanomesh serves as the substrate of the cells and is stretched across the sample holder containing collagen-I gel. The mesh is hold in place by a tightly fitting cone shaped ring.

Our skin equivalent (SE) construct models the epidermis and the connective tissue of the dermis with a layer of HaCaT keratinocytes and with a collagen gel, respectively. To provide a mechanical integrity suitable for experimental manipulations, we cultured the keratinocytes on the upper surface of an electrospun polycaprolactone (PCL) mesh, coated with fibronectin (Figure 1a). The median and mean fiber diameter of the mesh was 0.23±0.2 μm and 0.26±0.2 μm, respectively (Figure 1b). The mesh was stretched over the orifice of a short, 3D-printed sample holder tube (Figure 1c), which was filled with collagen-I gel (Figure 1d,e). The electrospun membrane was held in place by a plastic ring, allowing to remove the membrane and the cell layer for histological analysis at the conclusion of the experiments. The geometry of the sample holder device can be adjusted to fit various experimental conditions—our samples were cultured in 24-well plates and then inserted into microfluidic Franz chamber devices for epithelial transport measurements.

### 3.2. Histology of Skin Equivalents

**Figure 2:**
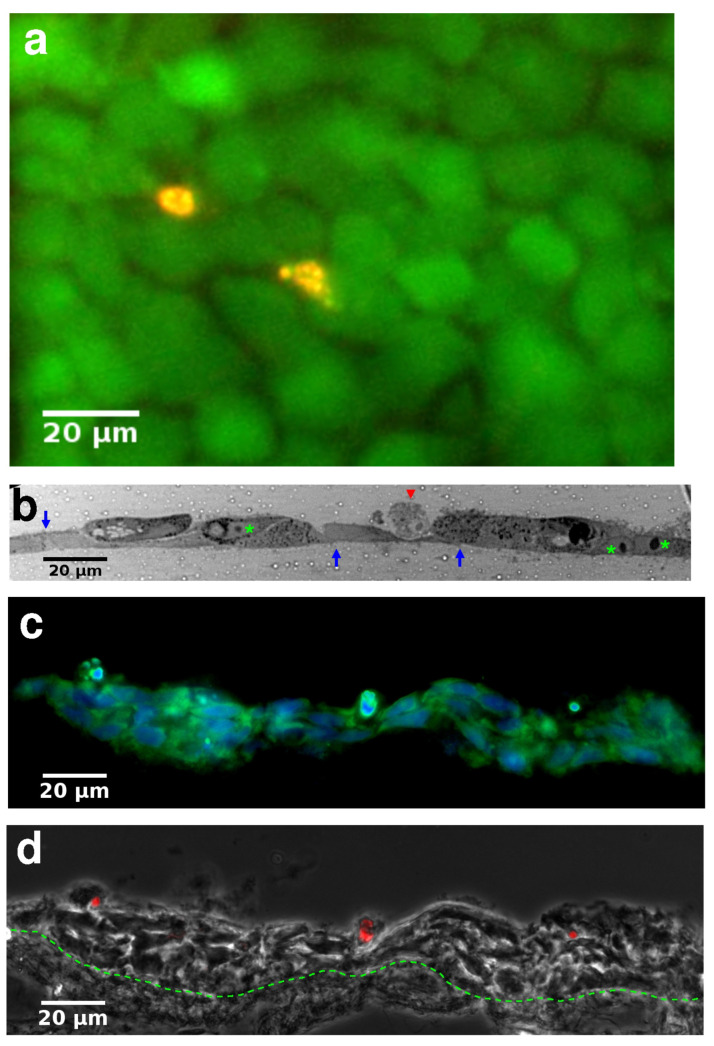
Histology of skin equivalents. (**a**) Live/dead labeled image of a monolayer of HaCaT keratocytes, imaged on the electrospun membrane at day 25 in culture. Calcein (green) fluorescence shows live cells while dead cell nuclei are identified by ethidium (red) fluorescence. Co-localization of green and red fluorescence appears as yellow. (**b**) Toluidine blue-stained semi-thin section of a monolayer of cells at day 18 in culture. Blue arrows point to cell-cell junctions, asterisks indicate cell nuclei with apparent nucleoli, red arrowhead marks an apoptotic cell. (**c**,**d**) Frozen cross-sections of a keratinocyte culture kept in culture for 28 days and at the air-liquid interface (ALI) for 14 days. (**c**) Cells and nuclei are visualized with calcein (green) and NucBlue (blue) fluorescence. (**d**) Phase-contrast image of the same field shown in (**c**) merged with ethidium fluorescence (red), identifying dead cells. Dotted line indicates the border between the cell layer and the electrospun membrane. Scale bars: 20 μm.

HaCaT keratocytes readily spread on the fibronectin-coated electrospun membrane surface and form a monolayer, as visualized by calcein/ethidium (“live/dead”) staining (Figure 2a). Semi-thin cross-sections reveal that the epithelium is composed of tightly connected cells (Figure 2b). When the epithelium-carrying membranes were brought to the air-liquid interface (ALI), cultures could be maintained for several weeks without substantial cell death. Frozen sections of the composite structure of the hydrogel, electrospun membrane and the epithelium reveal that in long-term ALI cultures HaCaT cells form stratified epithelia (Figure 2c,d).

### 3.3. Transepithelial Transport Measurements

**Figure 3:**
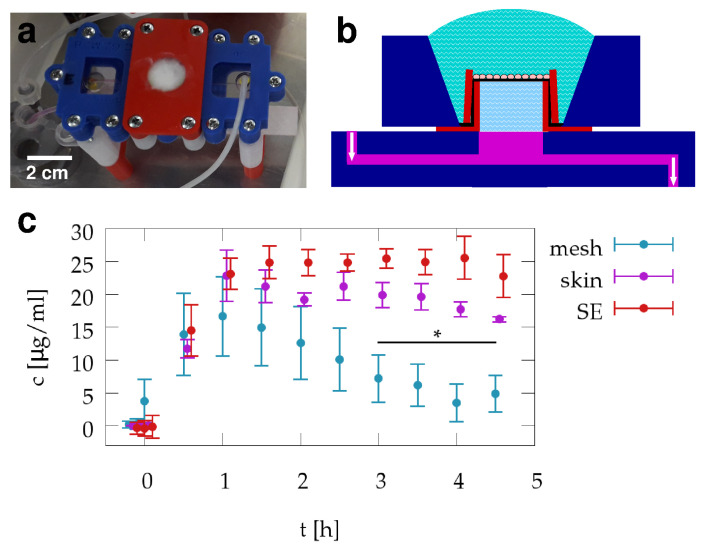
Transdermal transport measurements using skin equivalents and human skin. A photo (**a**) and schematic drawing (**b**) shows the sample holder device fitted into a microfluidic Franz chamber inside an incubator box. The cap of the chamber immobilized the sample holder device and held the caffeine-containing cream in close contact with the skin equivalent. Medium was transfused through the microfluidic chamber, and fractions of the medium leaving the chamber were collected every 30 min. (**c**) Caffeine concentration in the collected fractions was measured by spectrophotometry and is shown as a function of time. Caffeine exposition was started at t=0. Red, magenta and teal colored symbols represent the average caffeine concentration obtained from the skin equivalent, human skin and cell-free samples, respectively. Error bars represent SEM, from n=3 independent experiments. The difference between concentration readings from the marked mesh-only and SE samples is significant ($p=1.2\cdot 10^{-7}$, n=12 in each group).

For transdermal transport measurements we placed a microfluidic Franz chamber into a CO${}_{2}$ incubator box (Figure 3a). After establishing a steady flow of medium using a syringe pump, we positioned the skin equivalent sample above the microfluidic receptor cell and sealed the assembly with a cap that kept the epithelial layer exposed to the air and the potential pharmacological agents (Figure 3b). The transfused medium was collected and aliquoted into fractions every 30 min.

As a proof-of-concept transdermal transport experiment, we exposed skin equivalent samples, human skin preparations and cell-free PCL membrane-covered collagen samples to caffeine cream. We collected fractions both prior and after caffeine exposure. Spectrophotometric analysis of the fractions revealed the time-course of caffeine concentration in the acceptor chamber (Figure 3c). We found that caffeine concentration reached its maximal value one hour after exposing the samples to caffeine cream in all three samples. However, while the human skin and skin equivalent samples maintained this concentration without a substantial drop for up to five hours, passive diffusion through the cell-free sample yielded a concentration peak with a half-life of 2.5 h. We attribute this difference to the observation that the transfusion of the medium dissolved the cream in close proximity to the membrane. The ensuing gradual lack of close contact between the membrane and the cream reduced the transport efficiency of caffeine. Cells on the other hand, actively engaged the cream preventing the formation of gaps separating the cream from their apical surface.

## 4. Discussion

Electrospun meshes provide a highly customizable substrate for tissue engineering with very promising biological properties. In particular, the diameter of electrospun fibers is similar to that of fibrous extracellular matrix (ECM). Furthermore, the large surface area of electrospun meshes facilitates cell attachment and nutrient exchange. The utilized polymer and solvent system can have profound effects on the physical properties of the electrospun meshes [20,21]. Our choice, polycaprolactone (PCL), is a biochemically inert, US Food and Drug Administration (FDA)-approved material, widely used in slow release drug delivery devices [22] and as suture material [23]. While the hydrophobic nature of PCL hinders cell attachment, coating with ECM proteins (like collagen) provides suitable cell adhesion sites. From the several solvent systems that has been used for PCL electrospinning, we selected one that yields very thin nanofibers in a stable and reproducible manner [20]. PCL nanofiber meshes are rather stable: a pronounced change in its material and structural properties requires several months under standard tissue culture conditions [24]. These properties make electrospun PCL nanomeshes ideal candidates for skin equivalent engineering.

The PCL mesh can adsorb and release molecules [25,26], and electrospun fibers has been proposed as delivery devices for various anticancer drugs [27,28,29,30,31]. Importantly, caffeine release kinetics from PCL nanoparticles was recently reported to be rapid, reaching an equilibrium within an hour [32]. While this characteristic burst release of hidrophobic polymers [33,34] prompted research to combine electrospun fibers with nanoparticles exhibiting a slower release kinetics for drugs [35], in our experiments the time scale of transdermal transport is longer, and the caffeine creme was provided in a great excess. Thus, our results are unlikely to be affected by direct caffeine adsorption in the PCL mesh.

Over the past decade several attempts have been made to create human skin equivalents. Skin equivalents can model both the epidermis and dermis by combining isolated primary keratinocytes and fibroblast cells with hydrogels [36,37,38,39]. The similarity between skin equivalents and human skin samples was established by histological analyses and their functional integration was verified by implantation into immunodefficient mice [40]. Besides all its advantages, one of the known issues with this emerging tissue engineering technology is the insufficient mechanical properties of current skin equivalents. The low tensile strength of several biological hydrogels makes them difficult to use them in classic transdermal transport measurements within Franz diffusion cells [41,42]. To improve the poor mechanical properties various electrospun polymers can be used [43,44,45], however as yet we have not seen the utilization of this opportunity in skin equivalent engineering. Our approach proposed here combines electrospun poly-caprolactone substrate for improved mechanical stability of skin equivalents and microfluidic Franz diffusion cells for transdermal transport measurements.

The presented proof-of-concept study has several limitations. First, the epidermal and dermal layers of the skin are in reciprocal co-regulatory relationship. Keratinocytes of the epidermis influence fibroblasts of the dermal layer in a paracrine manner by producing IL-1, TNF-alpha, PDGF, TGF-beta and activin. Fibroblasts respond by producing extracellular matrix proteins and releasing KGF/FGF7, IL-6 and GM-CSF, which feeds back on the proliferation and differentiation of keratinocytes in a paracrine manner [46]. In this study we focused only on the epidermis and used serum in the culture medium to provide the survival factors for the keratinocytes. Further refinement of this technique can, however, also incorporate fibroblast cells in the collagen-I gel. A second limitation is that the hydrophilicity of the reagent is expected to have a large effect on transdermal transport, and we just investigated caffeine, a hydrophilic compound. The transdermal transport properties of caffeine are well documented, and the stratum corneum functions as the main diffusive barrier [47]. While the HaCaT cells in our skin equivalents were multi-layered in air-liquid interface cultures, they did not exhibit the characteristic hystology for the stratum corneum. We expect that primary keratocytes [36] or induced pluripotent stem cell-derived keratocytes [48] can overcome this limitation and form even better model for the epidermis. Finally, while the presented results demonstrate that transdermal transport through engineered skin equivalents can be readily measured using diffusion chambers, the establishment of quantitative equivalence requires further experiments with multiple compounds and adjustments in the culture conditions [49].

## Data Availability

All relevant data is included in the manuscript.
